# CD105 (Endoglin)-Negative Murine Mesenchymal Stromal Cells Define a New Multipotent Subpopulation with Distinct Differentiation and Immunomodulatory Capacities

**DOI:** 10.1371/journal.pone.0076979

**Published:** 2013-10-04

**Authors:** Per Anderson, Ana Belén Carrillo-Gálvez, Angélica García-Pérez, Marién Cobo, Francisco Martín

**Affiliations:** Department of Human DNA Variability, GENYO, Centre for Genomics and Oncological Research, Pfizer, University of Granada, Andalusian Regional Government, PTS Granada, Granada, Spain; Josep Carreras Leukaemia Research Institute, University of Barcelona, Spain

## Abstract

Administration of in vitro expanded mesenchymal stromal cells (MSCs) represents a promising therapy for regenerative medicine and autoimmunity. Both mouse and human MSCs ameliorate autoimmune disease in syn-, allo- and xenogeneic settings. However, MSC preparations are heterogeneous which impairs their therapeutic efficacy and endorses variability between experiments. This heterogeneity has also been a main hurdle in translating experimental MSC data from mouse models to human patients. The objective of the present manuscript has been to further characterize murine MSCs (mMSCs) with the aim of designing more efficient and specific MSC-based therapies. We have found that mMSCs are heterogeneous for endoglin (CD105) expression and that this heterogeneity is not due to different stages of MSC differentiation. CD105 is induced on a subpopulation of mMSCs early upon in vitro culture giving rise to CD105^+^ and CD105^-^ MSCs. CD105^+^ and CD105^-^ mMSCs represent independent subpopulations that maintain their properties upon several passages. CD105 expression on CD105^+^ mMSCs was affected by passage number and cell confluency while CD105^-^ mMSCs remained negative. The CD105^+^ and CD105^-^ mMSC subpopulations had similar growth potential and expressed almost identical mMSC markers (CD29^+^CD44^+^Sca1 ^+^ MHC-I^+^ and CD45^-^CD11b^-^CD31^-^) but varied in their differentiation and immunoregulatory properties. Interestingly, CD105^-^ mMSCs were more prone to differentiate into adipocytes and osteocytes and suppressed the proliferation of CD4^+^ T cells more efficiently compared to CD105^+^ mMSCs. Based on these studies we propose to redefine the phenotype of mMSCs based on CD105 expression.

## Introduction

Mesenchymal stromal cells (MSC) are non-hematopoietic, multipotent cells present in virtually all tissues and organs [1,2]. In vivo, MSCs are thought to be quiescent cells at a perivascular location which are mobilized upon injury in order to promote tissue repair [3]. In the bone marrow, MSCs constitute an important component of the hematopoietic stem cell niche and have been shown to support hematopoiesis both in vitro and in vivo [4,5]. Ex vivo cultured MSCs can readily be differentiated into cells of the mesodermal lineage such as ostocytes, chondrocytes and adipocytes and they possess potent immunomodulatory properties. These characteristics make them a promising tool for therapy in regenerative medicine, transplantation and autoimmune disease. Syn-, allo- and xenogeneic MSCs from various tissues have shown strong beneficial effects in many animal models of autoimmune disease [6]. Several clinical trials have been completed or are currently underway using human MSCs (hMSCs) for the treatment of autoimmune/inflammatory diseases [7–9]. Administration of MSCs is considered safe and well tolerated and the therapeutic effect in several early clinical trials is promising. However, the clear and consistent effects seen in numerous animal studies have not been achieved in clinical trials [10]. The explanations for this discrepancy may be many-fold. Firstly, the use of animal models poses limitations but also the differences found between murine MSCs (mMSCs) versus hMSCs, including phenotype and immunomodulatory mechanisms, could play part [11,12]. Secondly, ex vivo cultures of MSCs contain biochemically and phenotypically distinct cell types representing mesodermal progenitors at different stages of commitment as well as cells with no differentiation capacity. The transcriptional analysis of single cell-derived MSC colonies revealed the simultaneous expression of transcripts characteristic of various mesenchymal cell lineages, again suggesting that MSC preparations are not homogenous [13,14]. Thirdly, the use of MSCs for human disease relies on the injection of a large number of cells necessitating the expansion of MSCs in vitro for several weeks before yielding enough cells (>1-2 x 10^6^ cells/kg) [15,16]. However, the culturing of MSCs is associated with several problems including (i) loss of homing capacity due to down-modulation of chemokine receptor expression and increased cell size, (ii) increased susceptibility to cell death and (iii) loss of differentiation potential [17]. Therefore, a further characterization of mMSCs could help to understand the differences between mMSCs and hMSCs, to interpret the murine and human results and to develop improved therapeutic strategies using more standardized MSC preparations.

To date, no single MSC-specific marker exists and for hMSCs several minimal criteria have been proposed including (i) adherence to plastic, (ii) expression of CD73, CD90, CD105 and lack of CD31, CD34 and CD45 upon culture and (iii) capacity to differentiate into adipocytes, chondrocytes and osteocytes [11]. Mouse MSCs are similar to human MSCs but differ in phenotype, lacking or being heterogeneous for both CD73 and CD90 [18,19]. CD105 (endoglin) is a high affinity coreceptor for transforming growth factor (TGF)-β1 and TGF-β3 [20]. Although CD105 is generally considered an important marker for MSCs [11,21] several reports have shown that its expression vary depending upon MSC source (bone marrow-, adipose tissue-, umbilical cord blood derived MSCs or placenta-derived MSCs), culture time in vitro and differentiation state [22–25].

We found that CD105 expression on mMSCs is heterogeneous which is in agreement with previous studies on mMSCs [26,27] and on hMSCs [23,28–31]. Absence of CD105 expression (CD105^-^) on mMSCs and hMSCs have been shown to identify differentiated MSCs with increased osteogenic gene expression [26,30] while selection of CD105 positive (CD105^+^) MSCs favors chondrogenesis [32]. In spite of these results it is not clear whether this heterogeneity in CD105 expression is due to the presence of differentiated cells (and therefore with a limited expansion capability) or if they represent a distinct multipotent MSC subpopulation. In order to clarify this point, we set out to characterize the properties of CD105^-^ and CD105^+^ adipose tissue-derived murine MSCs (mASCs). We found that CD105^-^CD29^+^CD44^+^sca-1 ^+^ CD45^-^CD11b^-^ mASCs represent a distinct MSC subpopulation. Both CD105^-^ and CD105^+^ mASCs had similar proliferative capacity, colony-forming unit-fibroblast (CFU-F) potential and expression of differentiation-related genes and shared all other MSC markers analyzed. We found that CD105^-^ mASCs had greater capacity to differentiate into adipocytes and osteocytes compared to CD105^+^ mASCs. Interestingly, CD105^-^ mASCs were more efficient at inhibiting T cell proliferation in vitro.

## Materials and Methods

### Animals

Male Balb/c (6-10 weeks) and C57Bl/6 (6-8 weeks) (Charles River, Barcelona, Spain) were used to initiate cultures of mASCs or bone marrow-derived MSCs (BM-MSCs) or for the generation of splenocyte cell suspensions. All experiments were performed according to the Institutional Guidelines for the Care and Use of Laboratory Animals in Research and with the approval of the local ethical committee at the Hospital Virgen Macarena in Seville, Spain.

### Isolation and culture of murine mesenchymal stromal cells

Abdominal (epididymal) and subcutaneous (inguinal) fat from male Balb/c and C57Bl/6 mice (6-10 weeks or age; Charles River, Barcelona, Spain) was processed as previously described [33]. Briefly, fat tissue was aseptically removed, cut into small pieces, resuspended in 2.5 ml of Hank’s Balanced Salt Solution (HBSS, Invitrogen, Carlsbad, CA), containing 2 mg/ml collagenase type I (Sigma Aldrich, St. Louis, MO) per gram of fat tissue and incubated for 30 minutes at 37°C. After washing, cells were resuspended in complete MesenCult (Stem Cell, Grenoble, France) medium containing 20% mouse Mesenchymal Stem Cell Stimulatory Supplements (Stem Cell) and 100 U/ml penicillin/streptomycin (Invitrogen) and seeded at a density of 15,000-30,000 cells/cm^2^ and cultured at a 5% O_2_/5% CO_2_ atmosphere. Non-adherent cells were removed after 24 hours in culture. Subsequent passages were plated at 10,000 cells/cm^2^ in complete MesenCult medium. BM-MSCs were derived by flushing femurs and tibiae of Balb/c mice and adding 1.5 x 10^6^ cells/cm^2^ in cell culture flasks. BM-MSCs were used at passage >5.

### Purification and stimulation of CD105^-^ and CD105^+^ mASCs

For purification of CD105^+^ and CD105^-^ mASCs, mASCs (passage 2-4) were labeled with anti-CD105-PE antibodies (Abs) (eBioscience, San Diego, CA), subsequently labeled with anti-PE microbeads according to the manufacturer’s instructions (Miltenyi Biotech, Bergish Gladbach, Germany) and separated using an AUTO MACSpro cell separator (Miltenyi Biotech). The acquired cell populations were >90% pure. For stimulation with TGF-β1, cells were cultured for 24 hours in MesenCult with 1% supplements and then stimulated with 10 ng/ml recombinant human TGF-β1 (Peprotech, London, UK) for different time points. Total mASCs (ASC^tot^), CD105^-^ and CD105^+^ cells were stimulated with 10 ng/ml TNF-α and 10 ng/ml IFN-γ (Peprotech) for 12 and 24 hours for RNA extraction and 24 hours for flow cytometry. Cell confluence was quantified using the ImageJ software (http://rsbweb.nih.gov/ij/) and 25 µM GM6001 (Millipore, Billerica, MA) was added to the cells to inhibit matrix metalloproteinases (MMPs).

### ASC proliferation and colony forming unit-fibroblast (CFU-F) assay

Cell proliferation was assayed by seeding passage 3 ASC^tot^, CD105^-^ and CD105^+^ mASCs in T25 culture flasks at 2500 cells/cm^2^. Cells were harvested at 70-80% confluence, counted and reseeded at the same density for 10 passages. The CFU-F assay was performed as previously described [23]. In brief, ASC^tot^, CD105^-^ and CD105^+^ mASCs were seeded in 10 cm petri dishes at 2000 cells/plate and cultured for 14 days in complete MesenCult medium. Half of the media were replaced with fresh medium every 3-4 days. Cells were subsequently washed with PBS, fixed with 3.7% paraformaldehyde (15 minutes at room temperature) and stained with 1% cresyl violet. Colonies >2 mm were counted.

### Flow cytometry

Collagenase type I digests or cultured ASCs were incubated with 7AAD (Sigma-Aldrich) and 2.4G2 (eBioscience) and then stained with Abs specific for CD11b, CD29, CD31, CD34, CD44, CD45, CD49d, CD49f, CD61, CD73, CD105, CD140a, CD146, sca-1, MHC class I and MHC class II (all from eBioscience) or anti-TGF-βRI (ALK5) and anti-TGF-βRII Abs (R&D Systems, Minneapolis, MN). Cells were acquired and analyzed on a BD FACS Canto II using the FACS Diva software (BD Biosciences, Bedford, MA).

### MSC differentiation

ASC^tot^ and MACS-sorted CD105^-^ and CD105^+^ mASCs (passage 3-5) were plated in 6-well plates at a density of 20,000 cells/cm^2^ for adipogenesis, 10,000 cells/cm^2^ for osteogenesis or 0.5-1 x 10^6^ cells/15ml tube for chondrogenesis. MSC differentiation was induced using the hMSC adipogenic-, osteogenic- and chondrogenic BulletKits, (LONZA, Walkersville, MD) respectively, according to the manufacturer’s instructions. Cells were maintained in parallel in complete MesenCult as controls for spontaneous differentiation. Adipocytes were visualized on day 14 using OilRED O (Sigma-Aldrich). In brief, cells were fixed using 4% paraformaldehyde for 20 minutes at room temperature and then washed twice with PBS. The cells were preincubated with 60% propanol in distilled water and subsequently stained with 0.3% OilRED in 60% propanol for 10 minutes at room temperature. For quantification, cells were washed with PBS, air dried and the OilRED O extracted with isopropanol. Absorbance was measured at 450 nm. For osteogenesis, cells were stained with Alizarin RED (Sigma-Aldrich) on day 16 of differentiation. Stained area were quantified using the ImageJ software and is represented as % of total area. Chondrocyte pellets were fixed in 4% paraformaldehyde, embedded in paraffin and sections (5 µm) were stained with Alcian blue (pH 1).

### Quantitative PCR (qPCR)

The RNA from mASC was obtained using the trizol reagent (Invitrogen) according to the manufacturer’s instructions. RNA samples were reverse-transcribed using the Superscript First-Strand kit (Invitrogen) and qPCRs were performed using the QuantiTect SYBR, Green PCR kit (Qiagen, Valencia, CA) on a Stratagene MX3005P system. Primers: murine CD105S and CD105L transcripts were amplified using primer pairs as previously described: CD105L FW: 5´-GCACTCTGGTACATCTATTCTCACACACGTGG-3´; CD105L RV: 5´-GGGCACTACGCCATGCTGCTGGTGG-3´; CD105S FW: 5´-TGAGTATCCCAAGCCTCCACCCCAT-3´; CD105S RV: 5´-CTGAGGGGCGTGGGTGAAGGTCAG-3´ [34]; β-glycan FW: 5´-AGCTTCACCGTTCTGTCTGG-3´; β-glycan RV: 5´-AACTGGACCACAGAACCCTC-3´; ALK1 FW: 5´-GGCCTTTTGATGCTGTCG-3´; ALK1 RV: 5´-ATGACCCCTGGCAGAATG-3´; ALK2 5´-TTGCTCTCCCCTCCCCTA-3´; ALK2 RV: 5´-AGCACTGCTGGCCTTCAC-3´; ALK5 FW: 5´-TGTGCACCATCTTCAAAAACA-3´; ALK5 RV: 5´-ACCAAGGCCAGCTGACTG-3´; MMP-14 FW: 5´-GTGCCCTATGCCTACATCCG-3´; MMP-14 RV: 5´-CAGCCACCAAGAAGATGTCA-3´; ALP FW: 5´-TGTCATCATGTTCCTGGGAG-3´; ALP RV: 5´-ACACAAGTAGGCAGTGGCAG-3´; Osteocalcin FW: 5´-CTGACCCTGGCTGCGCTCTG-3´; Osteocalcin RV: 5´- GGCTGGGGACTGAGGCTCCA-3´; PPAR-γ FW: 5´-AATCCTTGGCCCTCTGAGAT-3´; PPAR-γ RV: 5´-TTTTCAAGGGTGCCAGTTTC-3´; LPL FW: 5´-TCCACCTCTTAGGGTACAGC-3´; LPL RV: 5´-AATGTCAACATGCCCTACTG-3´; SOX-9 FW; 5´-AAGACTCTGGGCAAGCTCTG-3´ SOX-9 RV: 5´-AGATCAACTTTGCCAGCTTG-3´; nanog FW: 5´-TTGCCTAGTTCTGAGGAAGC-3´; nanog RV: 5´-AACACAGTCCGCATCTTCTG-3´; iNOS FW: 5’-GTTCTCAGCCCAACAATACAAGA-3’; iNOS RV: 5’-GTGGACGGGTCGATGTCAC-3’; IL-6 FW: 5’-TAGTCCTTCCTACCCCAATTTCC-3’; IL-6 RV: 5’-TTGGTCCTTAGCCACTCCTTC-3’; IL-11 FW: 5´-TCCTTCCCTAAAGACTCTGG-3´: IL-11 RV: 5´-TTCAGTCCCGAGTCACAGTC-3´; β-actin FW: 5´-AATCGTGCGTGACATCAAAG-3´; β-actin RV: 5´-ATGCCACAGGATTCCATACC-3´.

### Assessment of suppressive activity of mASCs in vitro

ASCs were treated with mitomycin C (50 µg/ml, Sigma-Aldrich) for 20 minutes at 37°C and plated at different concentrations in flat bottomed 96-well plates and allowed to adhere for 3-4 hours. Splenocytes were labeled with 5 µM CFSE (Sigma-Aldrich) and anti-CD3 (1 µg/ml, BD Biosciences) was added to the cultures as a mitogenic stimulus of T cells. Cells were harvested on day 3, stained with anti-CD4-APC Abs and analyzed on a FACS Canto II flow cytometer (BD Biosciences). Cell division was analyzed using the FlowJo software (Tree Star Inc, Ashland, OR).

### Macrophage differentiation and cocultures

BM-derived macrophages (BM-MΦs) were generated as previously described [35]. In brief, 0.4 x 10^6^ BM cells/ml from Balb/c mice were cultured in DMEM (2 mM L-glutamine, 100 units/ml penicillin/streptomycin and 20% heat-inactivated FCS, all from Gibco/Invitrogen) containing 20 ng/ml M-CSF (Peprotech) for 5-7 days. Differentiated MΦs were detached by incubating the plates with PBS containing 2mM EDTA at 37°C for 10 minutes and gently flushing off the cells. For coculture experiments, ASC^tot^, CD105^-^ and CD105^+^ mASCs were added to 24-well plates (40,000 cells/well) with MΦs (0.15 x 10^6^ cells/well) and cultured for 48 hours at normoxia. In addition, MΦs and mASCs were added to wells separately as controls. The cells were then cultured for another 24 hours with or without LPS (B4:11; 1 µg/ml; Sigma-Aldrich) after which the supernatants were harvested and stored at -20°C for the quantification of IL-10 and IL-12 by ELISA.

### Measurement of PGE_2_ and cytokine production by mASCs

ASC^tot^, CD105^-^ and CD105^+^ mASCs (20,000 cells/cm^2^) were cultured with or without TNF-α and IFN-γ and the supernatants were collected after 24 and 48 hours. Cytokine content and PGE_2_ levels were analyzed using Ready-SET-go ELISA kits for TGF-β1, IL-10, IL-12 (eBioscience) or a PGE_2_ ELISA (Cayman Chemicals, Ann Arbor, MI), according to the manufacturer’s instruction. For measuring TGF-β1, complete MesenCult was added to cell-free wells, collected and frozen in parallel with supernatants from mASC containing wells. All samples were treated with 1M HCl and neutralized with 1M NaOH according to the manufacturer’s description in order to activate the TGF-β1. The plotted values were obtained by subtracting the TGF-β1 levels in the MesenCult medium from the cell culture supernatants.

### Statistical analysis

Statistical analysis was performed using the GraphPad Prism software (GraphPad Software, Inc, La Jolla, CA). All data are represented as mean (SEM) of 3 independent experiments unless otherwise stated in the figure legends. Comparisons of data obtained from ASC^tot^, CD105^-^ and CD105^+^ mASCs have been performed using the nonparametric Kruskal-Wallis test followed by the Dunn’s post test. P values <0.05 were considered statistically significant.

## Results

### Murine MSCs are heterogeneous for CD105-Long (CD105L) expression

**Figure 1:**
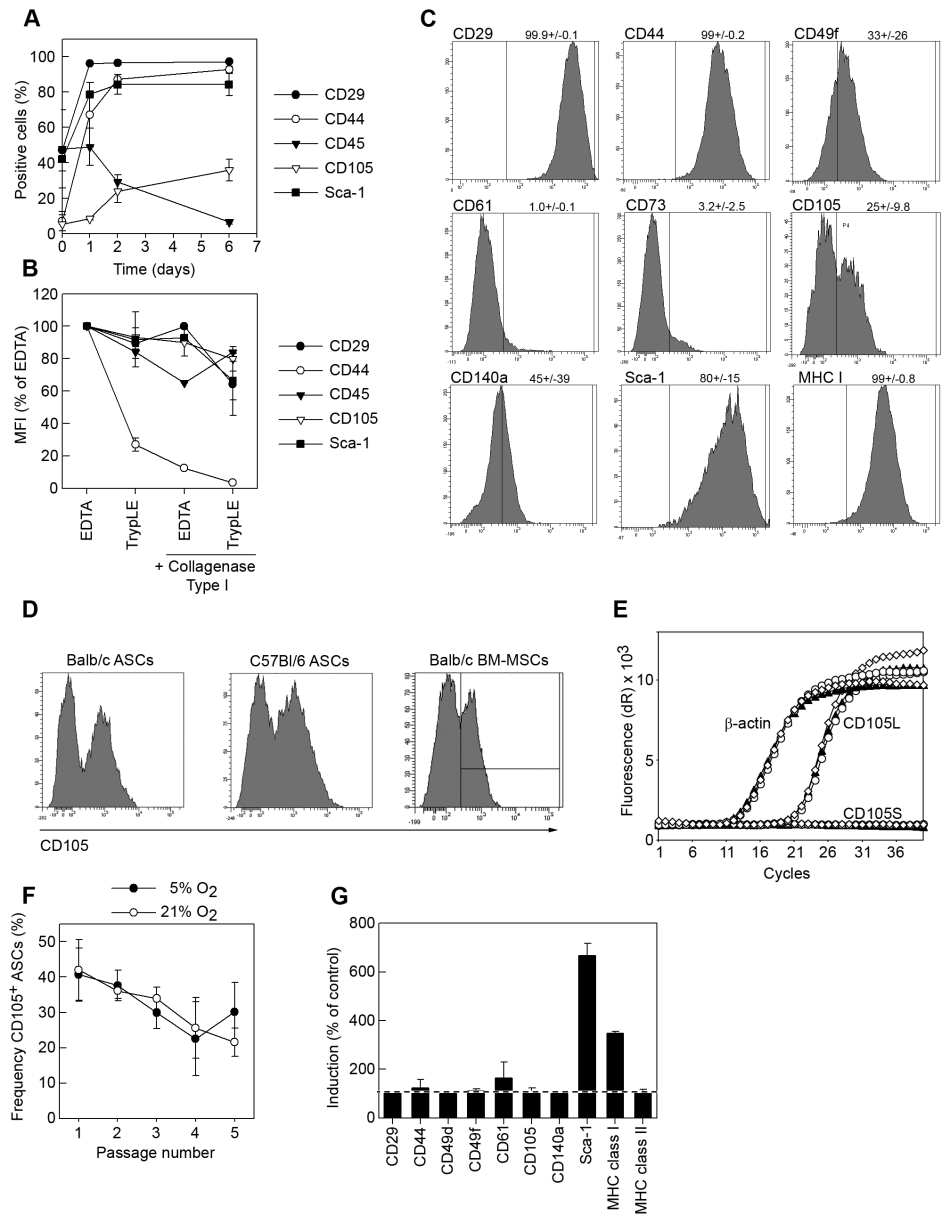
Murine MSCs are heterogeneous for CD105L expression. (A) Adipose tissue (subcutaneous and epididymal) from Balb/c mice were digested with collagenase type I for 30 minutes and the resulting cell suspension was stained for several cell surface markers before (day 0), 1, 2 and 6 days after plating on tissue-culture treated plastic and analyzed by flow cytometry. (B) ASC cultures were harvested with EDTA (2mM in PBS), TrypLE, or EDTA followed by treatment with collagenase type I or TrypLE followed by treatment with collagenase type 1 and analyzed by flow cytometry. The expression level (MFI) of each marker was normalized to the corresponding staining on EDTA-harvested cells. (C) Passage 4 mASCs were stained for a panel of surface antigens and analyzed by flow cytometry. Percentages of positive cells are represented as means (SEM) of 4 independent experiments. (D) ASCs from Balb/c and C57Bl/6 mice and BM-MSC from Balb/c mice were stained for CD105 and analyzed by flow cytometry. (E) Total RNA was purified from three independent mASC-cell preparations (passage 4-5), reverse transcribed and the expression of CD105L, CD105S and β-actin were analyzed by qPCR. (F) Total mASCs were cultured at either 5% O_2_ or 21% O_2_ for five passages and CD105 expression was analyzed for each passage by flow cytometry. (G) ASCs were cultured with or without TNF-α (10 ng/ml) and IFN-γ (10 ng/ml) for 24 hours after which the cells were harvested and analyzed by flow cytometry for a panel of MSC markers. Results in A, B and F are shown as mean (SEM) of 3 independent experiments; in C and D a representative experiment is shown out of at least 4 independent experiments; data in G is represented as mean (SEM) of 2 independent experiments.

In order to characterize the early phenotype of mASCs, we stained cell suspensions from collagenase type 1-digested adipose tissue (CD-AT) before and early after plastic adherence (1, 2 and 6 days of culture; before passage 1) for several MSC markers. Before plastic adherence, 35% and 50% of the cells expressed sca-1 and CD29, respectively, while they were almost negative for CD44 and CD105 (7% and 5% respectively) (Figure 1A). Upon in vitro culture at 5% O_2_, around 30-40% of all nucleated cells adhered to the plastic and this adherent fraction was nearly 100% positive for CD29, CD44 and sca-1 after 2 days in culture. In contrast, the CD105 expression reached a maximum of 30-40% after 6 days in culture. The contaminating CD45^+^ cells decreased rapidly during the initial expansion and had disappeared almost completely by day 6. The expression of CD44 was sensitive to both TrypLE and collagenase type 1 which can explain its absence on CD-AT. However, CD105 expression was resistant to the action of these enzymes (Figure 1B) and its absence on CD-AT cells suggests that it is induced on a subset of mASCs upon in vitro culture.

To further characterize the mASC cultures, we stained mASCs from Balb/c mice for a panel of standard MSC markers including several surface molecules with reported variation in the literature. FACS analysis showed that CD105 (endoglin) defined a positive (CD105^+^) and negative (CD105^-^) mASC population whereas the rest of the markers were homogenously expressed, yielding either positive (CD29, CD44, CD49f, CD140a and MHC class I) or negative (CD11b, CD45, CD49d, CD61, CD73, CD146 and MHC class II) ASC populations (data not shown; Figure 1C). The CD105^-^ mASCs did not represent CD105^low^ or CD105^intermediate^ ASCs since their CD105-staining overlapped completely with their corresponding isotype control (Figure S1). We found a similar CD105 expression pattern on MSCs derived from either adipose tissue or bone marrow independently of the strain used (Figure 1D, data not shown).

There exist two functional isoforms of CD105, CD105-Long (CD105L) and CD105-Short (CD105S) that have opposite effects on TGF-β signaling [36]. By qPCR we found that mASCs expressed the CD105L isoform whereas the CD105S isoform could not be detected (Figure 1E).

The differences observed in CD105 expression could be a consequence of culture conditions. We therefore investigated whether the expression of CD105 on mASCs could be modulated by culture time in vitro (passage number), cell-confluence, oxygen pressure or cytokines. We found a small but continuous reduction of CD105 expression during the expansion of mASCs and neither the initial CD105 expression levels nor the downregulation during passages were affected by the oxygen tension (Figure 1F; 21% O_2_, open circles; 5% O_2_, filled circles). TNF-α and IFN-γ are well known activators of MSCs [37] and have been shown to downregulate CD105 expression on vascular endothelial cells [38]. However, stimulating mASCs with both cytokines did not affect the CD105 expression while upregulating MHC class I and sca-1 expression on mASCs (Figure 1G).

**Figure 2:**
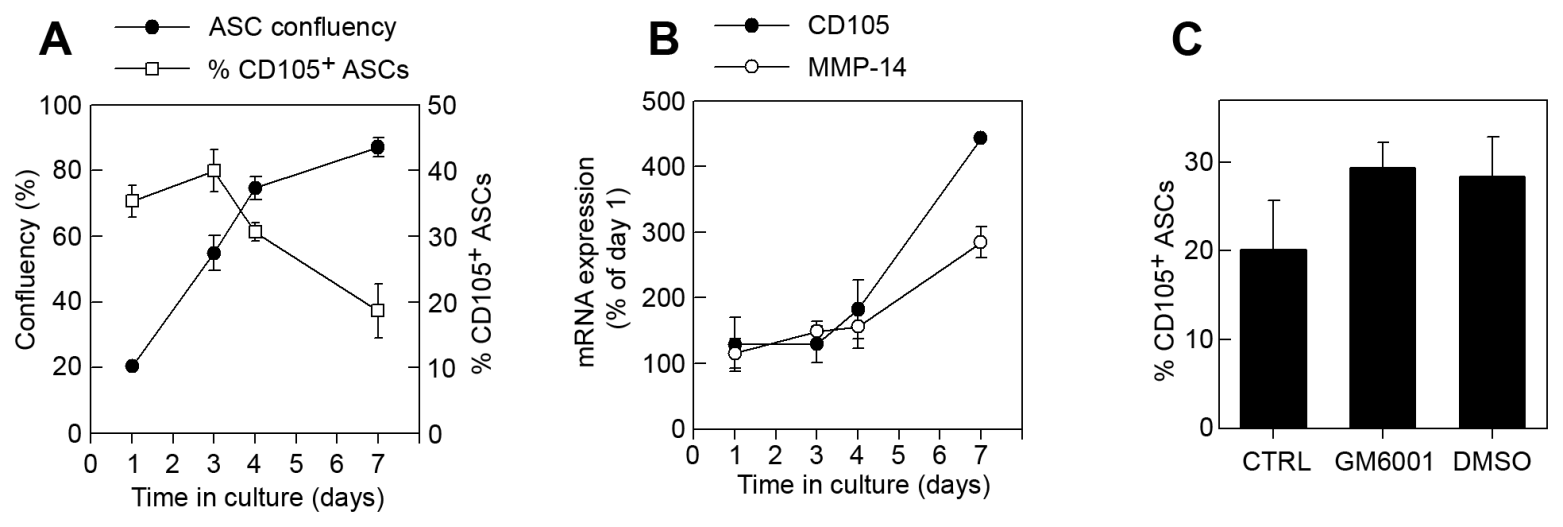
CD105 expression is downregulated on confluent mASCs. Total mASCs were plated at a low density (5000 cells/cm^2^) and grown to confluence during 7 days. Cells were harvested on day 1, 3, 4 and 7 and (A) CD105 expression was analyzed by flow cytometry and confluence was assessed using the ImageJ software and (B) total mRNA was purified, reverse transcribed and used for analyzing the expression levels of CD105 and MMP-14. (C) Total ASCs were seeded at 5000 cells/cm^2^ in 6 well plates and 4 days later, GM6001 (25 µM) or vehicle (DMSO) were added to the cells. Cells were harvested after 72 hours and CD105 expression was analyzed by flow cytometry. Data is shown as mean (SEM) of at least 3 independent experiments.

Finally, the degree of cell confluency has been shown to affect the expression of cell surface markers [39,40]. We found that the CD105 surface expression decreased when the mASC cultures became confluent while the CD105 mRNA levels increased (Figure 2A and 2B). It has recently been shown that membrane type 1 matrix metalloproteinase (MT1-MMP/MMP-14), which is a membrane-tethered MMP, can cleave CD105 from the cell surface [41]. We found that the MMP-14 mRNA increased when the mASC cultures reached confluency and addition of the MMP-inhibitor GM6001 at this stage increased the CD105 levels on mASCs. However, the vehicle control (DMSO) also increased CD105 expression to the same extent suggesting that MMP-14 is not responsible for the decrease in CD105 expression on mASCs (Figure 2B and 2C).

Taken together, these data shows that mMSCs are heterogeneous for CD105L and that its expression levels can be partially modified by the number of passages and the degree of cell confluency.

### CD105 defines two MSC subpopulations in mouse

**Figure 3:**
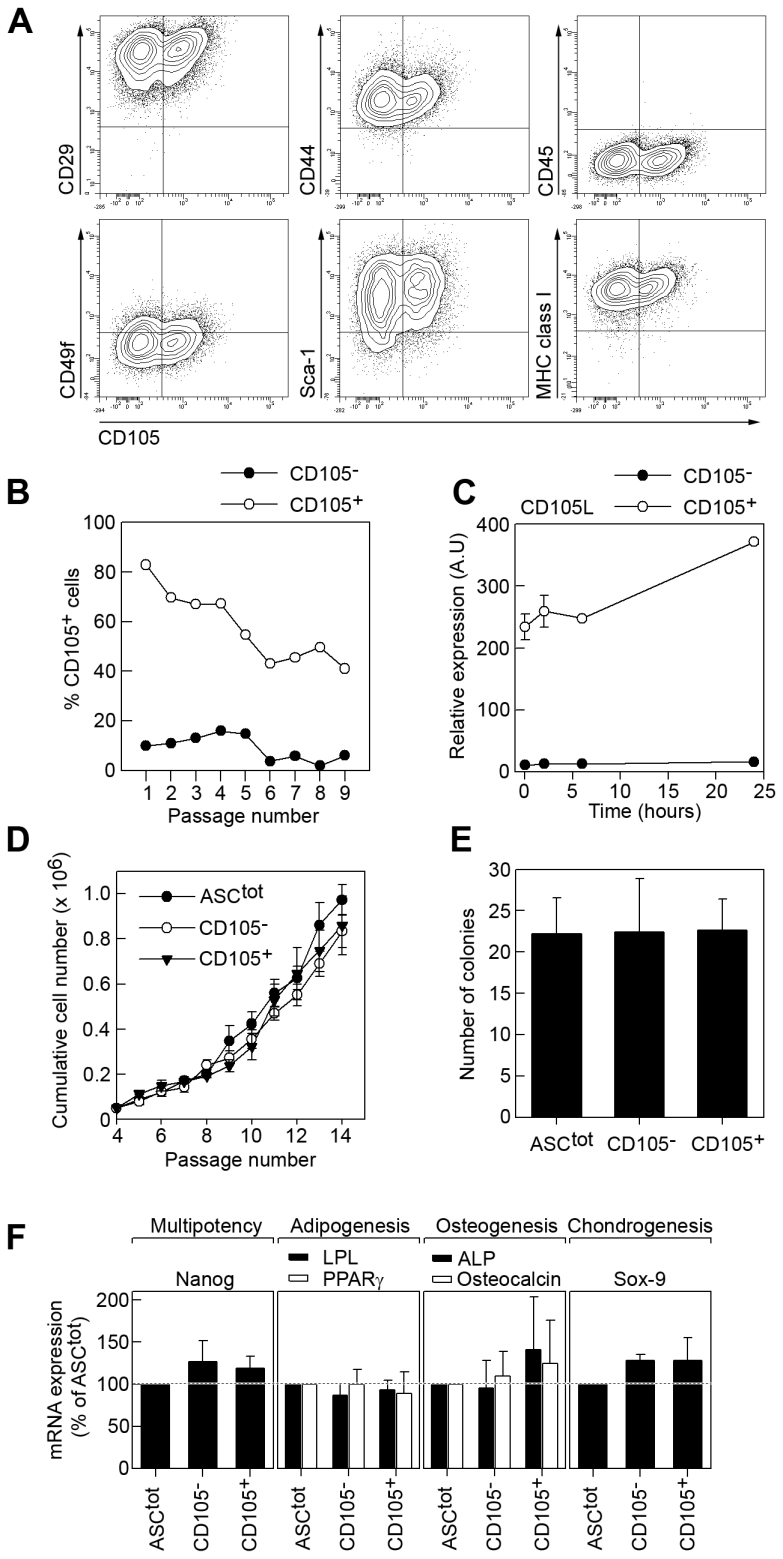
CD105 defines two distinct MSC subpopulations in mouse. (A) Balb/c ASCs were double-stained for CD105 in combination with CD29, CD44, CD45, CD49f, sca-1 and MHC class I and analyzed by flow cytometry. One representative experiment out of 3 independent experiments is shown. (B) MACS-purified CD105^-^ and CD105^+^ mASCs were cultured separately for 9 passages and CD105 expression was analyzed at each passage by flow cytometry. (C) CD105^-^ and CD105^+^ mASCs were starved for 24 hours and then stimulated with 10 ng/ml recombinant human TGF-β1 for 0, 2, 6 and 24 hours. At each time point total RNA was purified and reverse transcribed and the expression of CD105L was measured using qPCR. (D) ASC^tot^, CD105^-^ and CD105^+^ cells were plated at 2500 cells/cm in T25 cell culture flasks and cultured at 5% O_2_ until reaching 70-80% confluency. Cells were harvested, counted and reseeded at the same density for 10 passages. (E) ASC^tot^, CD105^-^ and CD105^+^ cells were cultured in 10 cm petri dishes (2000 cells/plate) for 14 days for CFU-F assay as described in materials and methods. Cells were subsequently fixed with PFA and stained with cresyl violet to visualize CFU-F. (F) Total RNA was extracted from ASC^tot^, and MACS-separated CD105^-^ and CD105^+^ mASCs and reverse transcribed into cDNA. qPCR analysis was performed on genes involved in ASC multipotency (nanog), adipogenesis (LPL, PPAR-γ), osteogenesis (osteocalcin, ALP) and chondrogenesis (Sox-9). Results are shown as mean (SEM) of three independent experiments.

The CD105^-^ and CD105^+^ mASCs could represent different stages of ASC activation and/or differentiation but could also represent multipotent subpopulations within the MSC-preparation with distinct properties. To investigate these possibilities we separated mASCs into CD105^-^ and CD105^+^ populations using magnetic-activated cell sorting (MACS). The resulting cell populations were routinely analyzed by flow cytometry (Figure S2A) and qPCR (Figure S2B). FACS analysis of the sorted populations showed that CD105^-^ and CD105^+^ cells shared a similar expression pattern of the characteristic MSCs markers: negative for CD45 and positive for CD29, CD44, CD49f, sca-1 and MHC class I (Figure 3A). Prolonged culturing of the sorted CD105^+^ mASCs resulted in a gradual loss of CD105 surface expression (Figure 3B, open circles), as observed in total populations (Figure 1F). Interestingly, the CD105^-^ population remained negative, indicating that CD105^-^ mASCs cannot acquire CD105 expression during culture (Figure 3B, filled circles). CD105^-^ cells were also unable to express CD105 upon addition of TGF-β1, while the CD105^+^ mASCs responded by slightly increasing their CD105 expression (Figure 3C).

These data suggest that CD105^-^ cells could represent a subpopulation of MSCs, distinct from the CD105^+^ cells. However downregulation of CD105 expression has been associated with MSC differentiation [24] and, in humans, the CD105 antigen has been used to select for multipotent MSCs [42]. We therefore studied the proliferation and clonogenicity of sorted CD105^-^ and CD105^+^ mASCs. If CD105^-^ cells represent differentiated cells they should have lower proliferative and clonogenic potential than CD105^+^ mASCs. Our studies demonstrated that there were no differences in cell size, proliferative or CFU-F capacity between the CD105^-^ and CD105^+^ ASCs (Figure S3, Figure 3D and 3E). In addition, we performed a qPCR screening of pluripotency- and lineage specific markers comparing total ASCs, CD105^-^ and CD105^+^ populations. We could not detect any differences in the expression of nanog (multipotency), PPAR-γ and LPL (adipogenesis), ALP and osteocalcin (osteogenesis) or Sox-9 (chondrogenesis) between the different mASC populations (Figure 3F). Also, we could not detect the expression of the preadipocyte marker pref-1 in any cell population (data not shown). Taken together, these data suggest that the CD105^-^ cells do not represent differentiated cells but rather a distinct MSC population.

### CD105^-^ mASCs are more prone to differentiate into adipocytes and osteocytes

**Figure 4:**
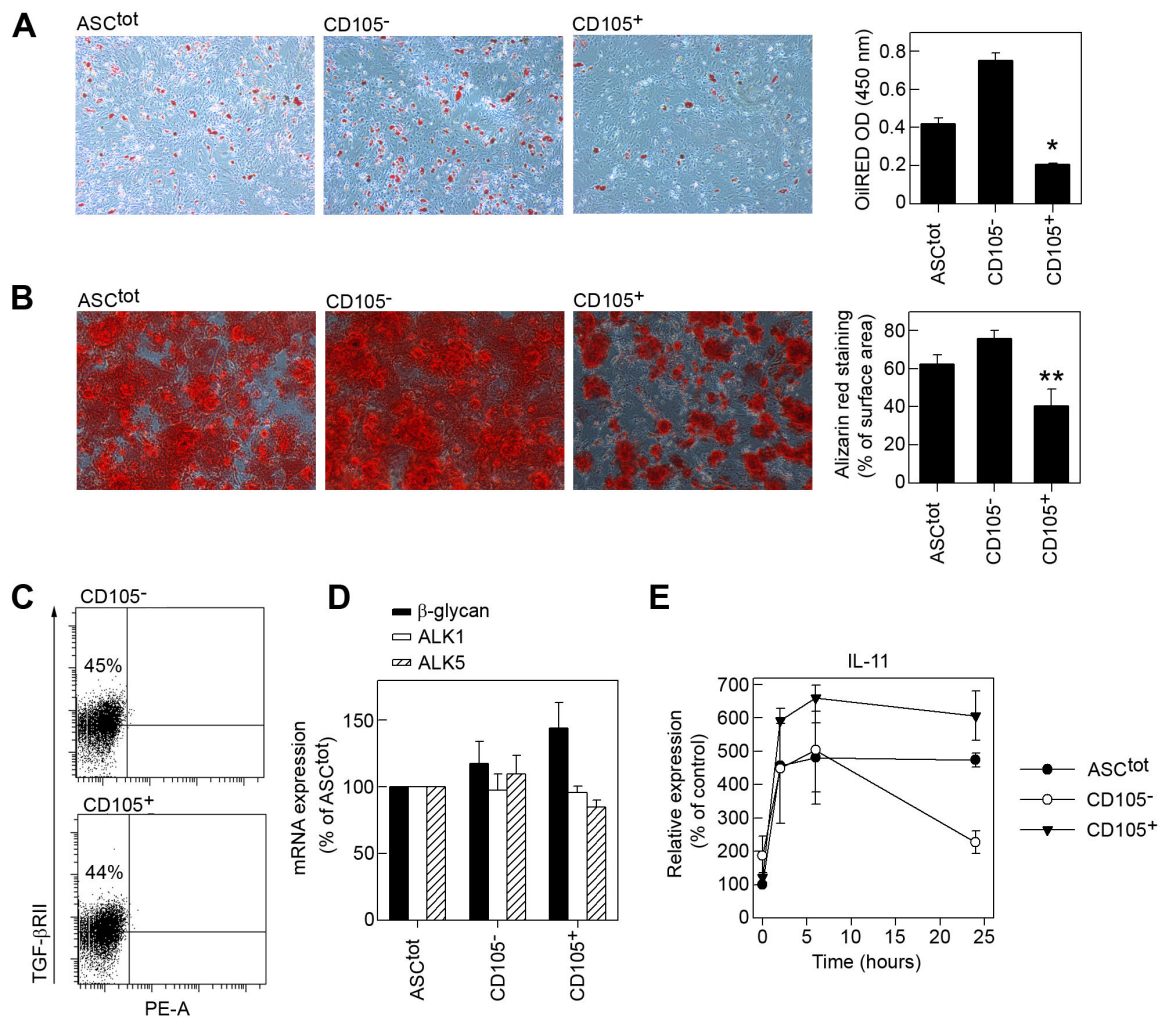
CD105^-^ ASCs are more prone to differentiate into adipocytes and osteocytes. Adipogenesis (A) and osteogenesis (B) were induced in ASC^tot^, CD105^-^ and CD105^+^ mASC populations. Differentiation data is shown as one representative experiment out of 3-4 independent experiments. The extent of adipocyte differentiation was visualized using OilRED O and the amounts of OilRED O extracted from the different ASC populations were measured using a spectrophotometer (450 nm). Bars are shown as mean (SD) from experimental triplicates. *=p<0.05 vs. CD105^-^ mASCs. The deposition of calcium by differentiating osteocytes was visualized using alizarin red and quantified using the ImageJ software. Bars represent mean (SD) from experimental triplicates. **=p<0.01 vs. CD105^-^ mASCs. (C) CD105^-^ and CD105^+^ mASC were stained for TGF-β receptor II (TGF-βRII) and analyzed by flow cytometry. (D) Total RNA was purified from ASC^tot^, CD105^-^ and CD105^+^ mASCs, reverse transcribed and the expression levels of β-glycan, ALK1, ALK2 and ALK5 assayed using qPCR. (E) ASC^tot^, CD105^-^ and CD105^+^ mASCs were starved for 24 hours and then stimulated with 10 ng/ml recombinant human TGF-β1 for 0, 2, 6 and 24 hours. At each time point total RNA was purified and reverse transcribed and the expression of IL-11 was measured using qPCR. Results are shown as mean (SEM) or two independent experiments. Expression values are plotted relative to ASC^tot^ 0h.

One of the defining characteristics of MSCs is their ability to differentiate into adipocytes, osteocytes and chondrocytes. We thus set out to investigate the differentiation capacity of CD105^-^ and CD105^+^ mASCs. We found that CD105^-^ cells generated significantly more adipocytes and osteocytes compared to total and CD105^+^ mASCs (Figure 4A and 4B). However, we did not find any significant difference in chondrogenesis between the different subpopulations (Figure S4).

As shown in Figure 3F the enhanced induction of CD105^-^ mASC into adipocytes and osteocytes cannot be attributed to the presence of cells already committed to become adipocytes (preadipocytes) and/or osteocytes (osteoprogenitor). TGF-β1 inhibits adipogenesis and osteogenesis [43] and therefore a reduction in TGF-β1 signaling could enhance differentiation towards these lineages. A reduction of CD105 or other TGF-β receptors on the ASCs could thus favor adipo- and osteogenesis. To analyze this possibility we first set out to study the expression pattern of different TGF-β receptors on CD105^-^ and CD105^+^ mASCs. We showed that both mASC subsets expressed similar levels of β-glycan, ALK1, ALK5 and TGF-β receptor II, while ALK2 was not expressed by either cell population (Figure 4C and 4D). Therefore, in principle, any difference in the TGF-β response between CD105^-^ versus CD105^+^ mASCs could be due to presence or absence of CD105. This is in agreement with the previously published papers where downregulation of CD105 enhanced osteogenic [30] and adipogenic [23] differentiation.

To further study the role of TGF-β signaling on ASC differentiation we measured the expression of interleukin-11 (IL-11), previously known as adipogenesis inhibitory factor (AGIF) [44]. IL-11 is a TGF-β1-inducible gene, dependent upon the activation of smad2/3/4. Stimulation of the different subsets with TGF-β1 revealed a higher and more sustained induction of interleukin-11 expression in CD105^+^ ASCs compared to CD105^-^ ASC (Figure 4E). The lower IL-11 expression by CD105^-^ ASCs upon TGF-β1 stimulation could partly explain the increased adipogenic capacity of this subset.

### CD105^-^ mASCs have improved immunomodulatory capacity in vitro compared to CD105^+^ mASCs

**Figure 5:**
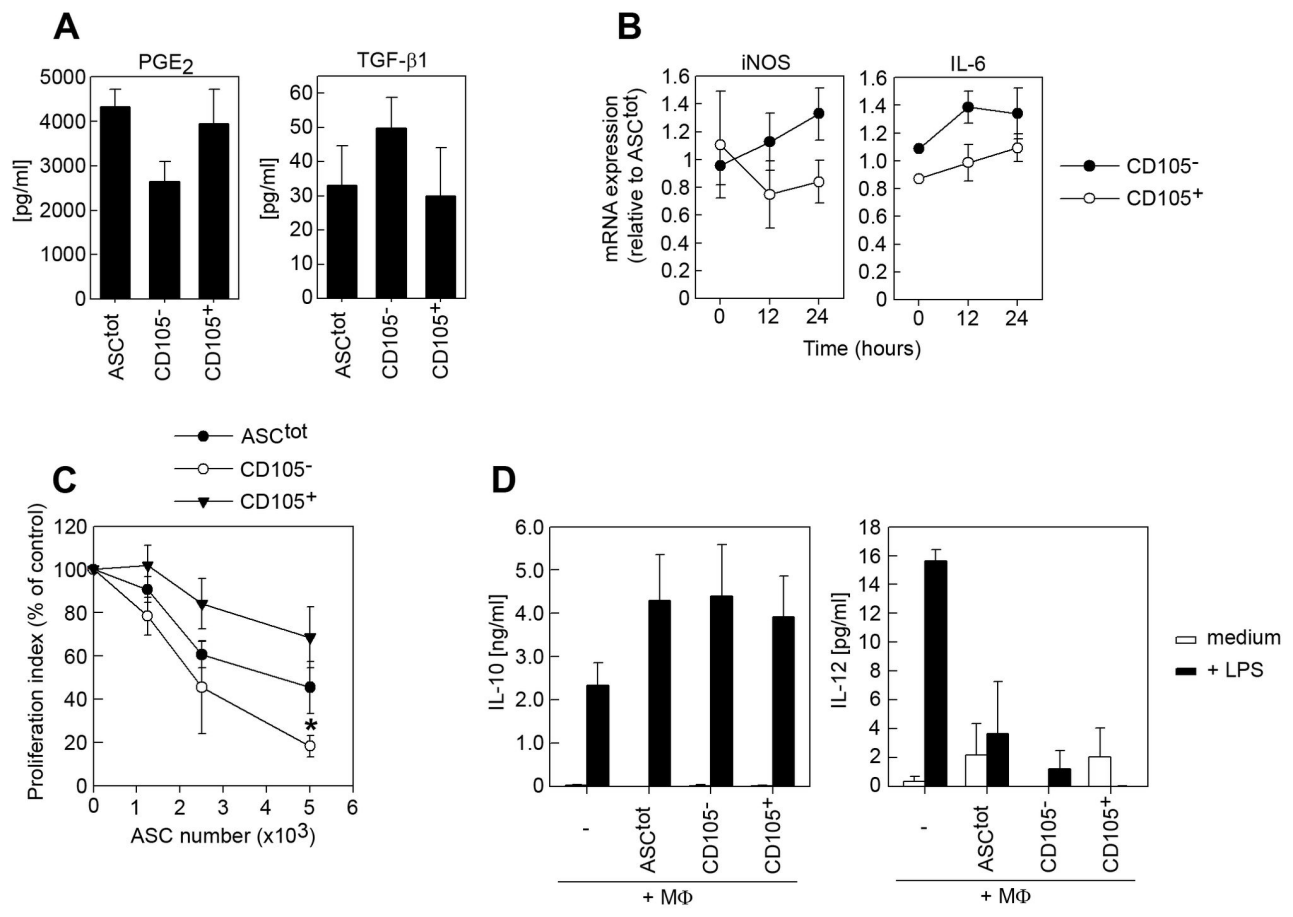
Comparison of the immunomodulatory capacity of CD105^-^ and CD105^+^ mASCs. (A) The levels of PGE_2_ and TGF-β1 were measured in supernatants from ASC^tot^, CD105^-^ and CD105^+^ mASCs using specific ELISAs (see materials and methods). (B) ASC^tot^, CD105^-^ and CD105^+^ mASCs were stimulated with TNF-α (10 ng/ml) and IFN-γ (10 ng/ml) for 12 and 24 hours. Total RNA was purified for each time point and the expression of iNOS and IL-6 was analyzed using qPCR. (C) Increasing numbers of mitomycin C-treated ASC^tot^, CD105^-^ and CD105^+^ mASCs were cultured together with CFSE-labeled splenocytes (200,000 cells/well) and stimulated with anti-CD3 (1 µg/ml) for 3 days. The cells were harvested and acquired on a FACS Canto II flow cytometer and the proliferation of CD4^+^ splenocytes was quantified using the FlowJo software. (D) BM-MΦs were cultured with or without mASC for 48 hours and then restimulated with LPS (1 µg/ml) for 24 hours. The levels of IL-10 (left graph) and IL-12 (right graph) in the supernatants of the co-cultures were measured using specific ELISAs. Data is shown as mean (SEM) of at least 3 independent experiments. *=p<0.05 vs. CD105^+^ mASCs.

Mesenchymal stromal cells are known to inhibit the immune response in vitro and in vivo through multiple mechanisms [3]. However, it is not known whether CD105^-^ and CD105^+^ mASCs are equally efficient in inhibiting the immune response in vitro. To this end we firstly measured the production of some important ASC-associated immunomodulatory molecules by the three cell populations. We found that CD105^-^ mASCs produced slightly more TGF-β1 and less PGE_2_ compared to ASC^tot^ and CD105^+^ mASCs but the differences were not statistically significant (Figure 5A). Stimulating the mASCs with TNF-α and IFN-γ resulted in a slightly higher induction of iNOS and IL-6 mRNA in CD105^-^ mASCs compared to CD105^+^ cells (Figure 5B). Both these mediators have been implicated in the immunomodulatory capacity of MSCs [45,46], including the inhibition of T cells. In agreement, we found that CD105^-^ mASCs were significantly better at inhibiting splenocyte proliferation compared to CD105^+^ mASCs (Figure 5C) at low ASC/splenocyte ratios.

We and others have shown that MSCs can induce a regulatory activation state in macrophages characterized by a high IL-10/IL-12 ratio upon restimulation [47–49]. Importantly, these regulatory macrophages (MΦregs) participate in the MSC-mediated suppression of T cell proliferation [48]. In order to assess the relative capacity of ASC^tot^, CD105^-^ and CD105^+^ mASCs to induce MΦregs, we cultured BM-MΦs with or without the different mASC populations for 72 hours in the presence of LPS for the last 24 hours. As previously described, we found that mASCs cocultured with activated macrophages resulted in an increase in the IL-10/IL-12 cytokine ratio suggesting a shift from classically activated macrophages to a regulatory phenotype. However, there was no difference between the ASC^tot^, CD105^-^ and CD105^+^ mASC in their capacity to induce MΦregs (Figure 5D).

## Discussion

The aim of the current study has been to characterize murine ASCs preparations based on cell surface markers and assess the in vitro differentiation potential and immunosuppressive capacity of any identifiable populations. The discovery of MSC subpopulations with increased self-renewal capacity, differentiation potential and/or immunomodulatory effects would allow us to design more specialized MSC therapies and, in addition, the usage of better defined MSC-preparations would make inter-study comparisons more straight forward. In the present manuscript we have defined two mASC populations based on expression of CD105, a component of the TGF-β receptor complex. The CD105^-^ mASCs exhibited improved differentiation and immunomodulatory capacities compared to total and CD105^+^ mASCs while sharing all other phenotypic, proliferative and clonogeneic properties.

CD105 is fundamental for the development and function of the cardiovascular system and has been implicated in angiogenesis [50], regulating the migration [51], survival and cytoskeletal organization [52] of endothelial cells. CD105 has generally been used to select for multipotent human MSCs and the disappearance of CD105 has been associated with MSC differentiation [24,26]. The impact of CD105 expression by MSCs on their therapeutic effect has been described in a few recent reports. Firstly, human CD105^+^ MSCs have been shown to be more efficient in repairing the infarcted heart [22]. Secondly, CD105^+^ hASCs were shown to be more prone to differentiate into chondrocytes compared to CD105^-^ hASCs [23,53] whereas CD105^-^ hASCs were more osteogenic [30].

Since CD105 is a component of the TGF-β receptor, its presence or absence on the ASCs must have an effect on their response to TGF-β. Firstly, MSC constitutively secrete TGF-β1 in culture and the fetal bovine serum contains high levels of latent TGF-β1 ( [54]; our unpublished observations) and secondly, TGF-β exerts important effects on MSCs differentiation [43,55] and immunomodulation [56]. Therefore, the responsiveness of MSCs to TGF-β will have an important impact on their physiology and therapeutic applications. As a modulator of TGF-β signaling, CD105 can either increase or decrease the ASC’s responsiveness to TGF-β1/3. CD105 exists in two isoforms, CD105L and CD105S, which play opposite roles in modulating TGF-β1/3-induced signaling [36]. We found that mASCs expressed only the CD105L isoform. CD105L inhibits ALK5/smad3 activation while it promotes ALK1/smad1 and ALK5/smad2 activation [30,57–59]. TGF-β-mediated activation of smad2 has been shown to inhibit both adipogenesis [60] and osteogenesis [30]. In agreement with this, we showed that CD105^-^ mASCs exhibited an enhanced adipo- and osteogenic potential, probably due to a reduced TGF-β/smad2 signaling. In addition, adipogenesis could be further promoted in CD105^-^ ASCs by the lower production of IL-11 upon TGF-β1 stimulation. Previous studies on the effect of CD105 on adipogenesis and osteogenesis using human ASCs have yielded contradictory results. Levi and colleagues showed that human CD105^low^ ASCs exhibited an enhanced differentiation into osteocytes but reduced adipogenic potential [30]. Possible explanations for the discrepancy between our data and those by Levi et al. with respect to the adipogenic potential of CD105^-^ ASCs could be species differences between mouse and human ASC or that the CD105^low^ hASC population represents osteoprogenitors with reduced adipogenic potential.

TGF-β could also act on the MSCs to modulate their immunosuppressive capacity. We found that CD105^-^ mASCs were more effective at inhibiting CD4^+^ T cell proliferation compared to CD105^+^ cells. While the mechanisms behind this difference are not well understood, we showed that CD105^-^ mASCs produced slightly more iNOS and IL-6 mRNA upon stimulation compared to CD105^+^ mASCs. In agreement with our data in MSCs, TGF-β1 has been shown to inhibit the production of iNOS/NO and IL-6 by macrophages and smooth muscle induced by LPS, IL-1, TNF-α and IFN-γ [61,62]. Therefore, in a MSC: splenocyte coculture, TGF-β1 could act more potently on CD105^+^ MSCs, reducing the production of iNOS and IL-6, and thus lowering their immunosuppressive activity. Macrophages are another important player in the MSC-mediated suppression of T cells [48]. However, despite a lower production of PGE_2_ by CD105^-^ ASCs, these cells were as efficient in inducing MΦregs as ASC^tot^ and CD105^+^ ASCs.

In addition to the multipotent CD105^-^ mASC population described in this manuscript, MSCs cultures can contain other CD105^-/low^ cells which originate from the CD105^+^ ASCs. The progressive loss of CD105 expression during culture has been reported using both mouse and human MSCs [22,63] and in agreement with these publications, we observed a progressive downregulation of CD105 expression on total and CD105^+^ ASCs that was not affected by culture conditions. Interestingly, we also found that the CD105 surface levels fluctuated during each passage depending upon confluence. Indeed, CD105, decreased when the ASC culture started to become confluent. This is in agreement with a study by Fonsatti and colleagues who showed that CD105 is a cell-density and proliferation dependent marker of endothelial cells [39]. Thus, a decrease in MSC cell proliferation, either due to culture confluence, senescence induction or spontaneous differentiation, could partly explain the loss of CD105 expression on MSCs.

Regardless of the mechanisms, based on our and others data, the lack/low expression of CD105 on ASCs due to heterogeneity or downregulation in culture might (i) decrease chondrocyte differentiation, (ii) increase adipogenesis/osteogenesis, (iii) decrease angiogenic capacity and (iv) affect their immunomodulatory capacity. Thus, when CD105 expression is of importance for the therapeutic efficacy of the MSCs, cell confluence, and culture time should be carefully controlled. In contrast, for immunomodulation, a lower expression of CD105 might be favorable. Although this study has been made using murine ASC we have obtained several results which are shared by human ASCs such as (i) downregulation of CD105 expression upon prolonged in vitro culture and (ii) the inhibitory effect of CD105 on osteogenesis. This suggests that our murine system is valid for the identification of general characteristics of ASCs that can be important for their therapeutic efficacy when treating human disease. In this direction, since in most cases hMSCs are CD105^+^, one should consider to select for CD105^+^ mMSCs when studying their effects in mouse models, in order to mimic the clinical settings.

In conclusion, we have identified a new murine CD105^-^ ASC subpopulation that has similar phenotype, proliferative capacity and CFU-F potential as CD105^+^ ASCs. Interestingly, CD105^-^ mASCs had greater capacity to differentiate into adipocytes and osteocytes compared to CD105^+^ ASCs and were better at inhibiting T cell proliferation in vitro. Although the isolation of CD105^-^ population from humans is more challenging due to the disappearance of these cells soon after culture initiation, it would be interesting to study if, by changing culture conditions and/or tissue of origin, we could obtain a similar, highly proliferative and homogenous human CD105^-^ MSC population.

## Supporting Information

Figure S1 **CD105 staining of mASCs reveals CD105^-^ and CD105^+^*subpopulations*.** Murine ASCs were stained with anti-mouse CD105-PE (0.1 µg/staining) and with its corresponding isotype control and analyzed by flow cytometry.
(TIF)

Figure S2 **CD105^-^ mASCs express lower levels of CD105 mRNA compared to CD105^+^ mASCs.** (A) mASCs were separated using magnetic activated cell sorting (MACS) into CD105^-^ and CD105^+^ cell populations. (B) Total RNA was purified from each cell populations, reverse transcribed and the expression levels of CD105L were analyzed using qPCR. Results are shown as mean (SEM) of 3 independent experiments. *=p>0.05 vs. CD105^+^ mASCs.
(TIF)

Figure S3 **Cell complexity of mASC subpopulations.** Murine ASCs were separated into CD105^-^ and CD105^+^ cell populations using magnetic activated cell sorting (MACS). The cell size (FSC) and granularity (SSC) of ASC^tot^, CD105^-^ and CD105^+^ mASCs were analyzed on a FACS Canto II flow cytometer. Results are shown as mean (SEM) of 4 independent experiments.
(TIF)

Figure S4 **Chondrogenic potential of CD105^-^ and CD105^+^ mASCs.** Total ASCs or sorted CD105^-^ and CD105^+^ mASCs (1 x 10^6^ cells/pellet) were resuspended in chondrogenic medium with TGF-β3 (20 ng/ml) and centrifuged to from a pellet. The medium was changed every 2-3 days. After 21 days, the pellets were fixed in 4% paraformaldehyde, embedded in paraffin, sectioned and stained for alcian blue.
(TIF)
