# Supplementary figures and images for: CD105 (Endoglin)-Negative Murine Mesenchymal Stromal Cells Define a New Multipotent Subpopulation with Distinct Differentiation and Immunomodulatory Capacities
Source: PLoS One. 2013 Oct 4;8(10):e76979. doi: 10.1371/journal.pone.0076979 (PMC3790740; doi:10.1371/journal.pone.0076979)

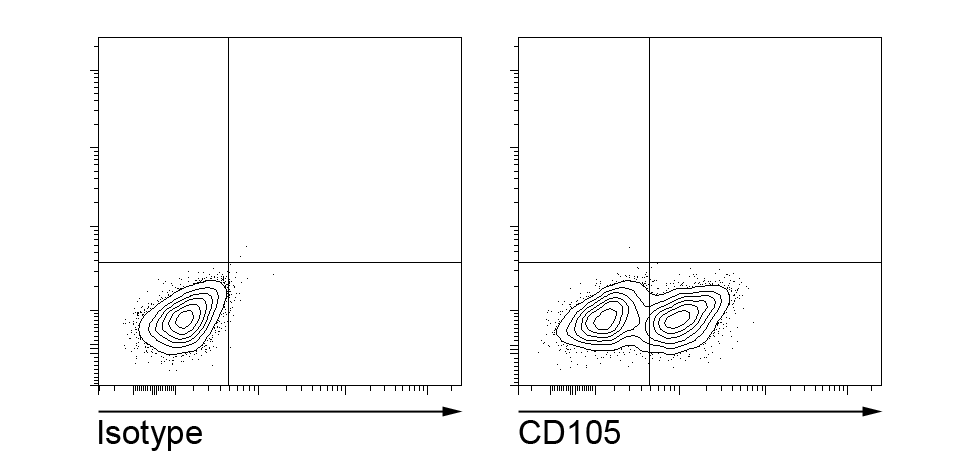

Supplement: Figure S1 — CD105 staining of mASCs reveals CD105- and CD105+subpopulations. Murine ASCs were stained with anti-mouse CD105-PE (0.1 µg/staining) and with its corresponding isotype control and analyzed by flow cytometry. (TIF) [file pone.0076979.s001.tif]

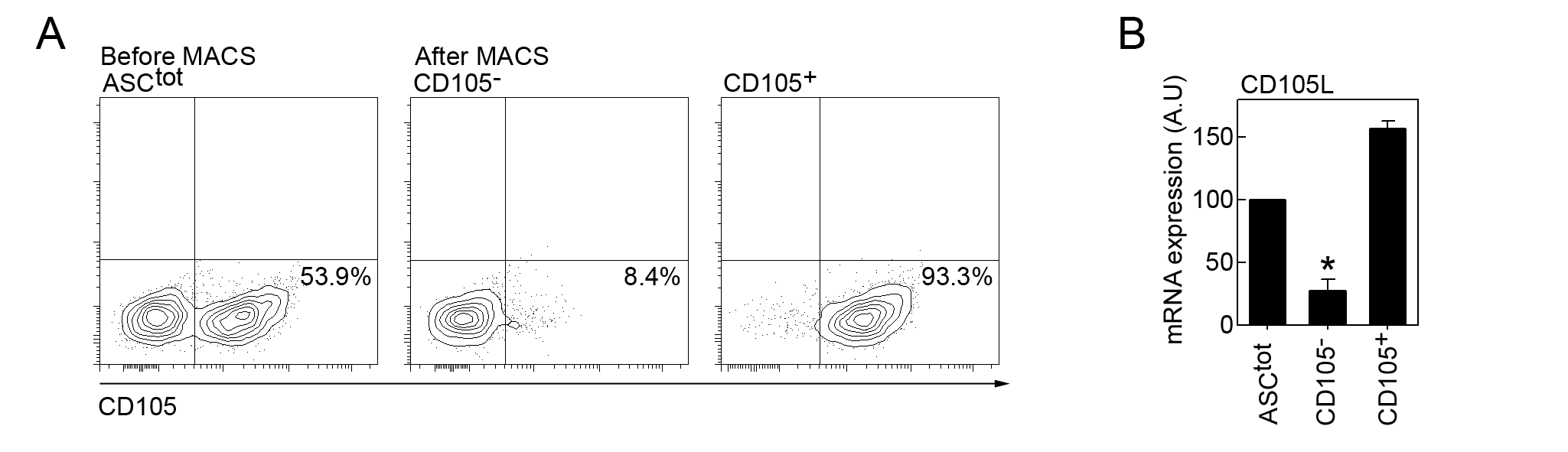

Supplement: Figure S2 — CD105- mASCs express lower levels of CD105 mRNA compared to CD105+ mASCs. (A) mASCs were separated using magnetic activated cell sorting (MACS) into CD105- and CD105+ cell populations. (B) Total RNA was purified from each cell populations, reverse transcribed and the expression levels of CD105L were analyzed using qPCR. Results are shown as mean (SEM) of 3 independent experiments. *=p>0.05 vs. CD105+ mASCs. (TIF) [file pone.0076979.s002.tif]

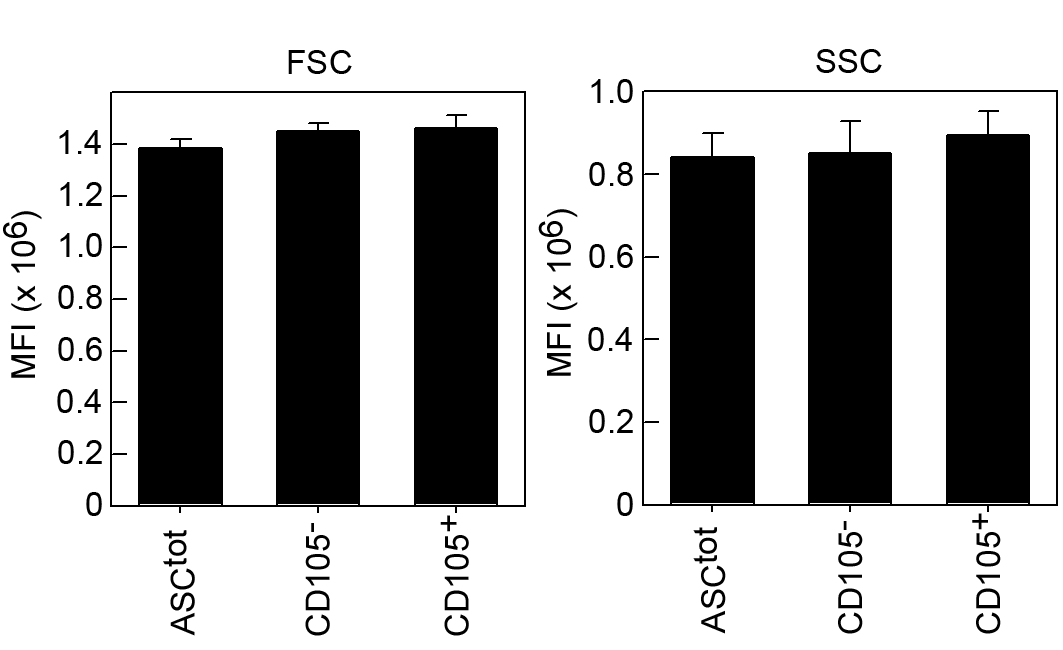

Supplement: Figure S3 — Cell complexity of mASC subpopulations. Murine ASCs were separated into CD105- and CD105+ cell populations using magnetic activated cell sorting (MACS). The cell size (FSC) and granularity (SSC) of ASCtot, CD105- and CD105+ mASCs were analyzed on a FACS Canto II flow cytometer. Results are shown as mean (SEM) of 4 independent experiments. (TIF) [file pone.0076979.s003.tif]

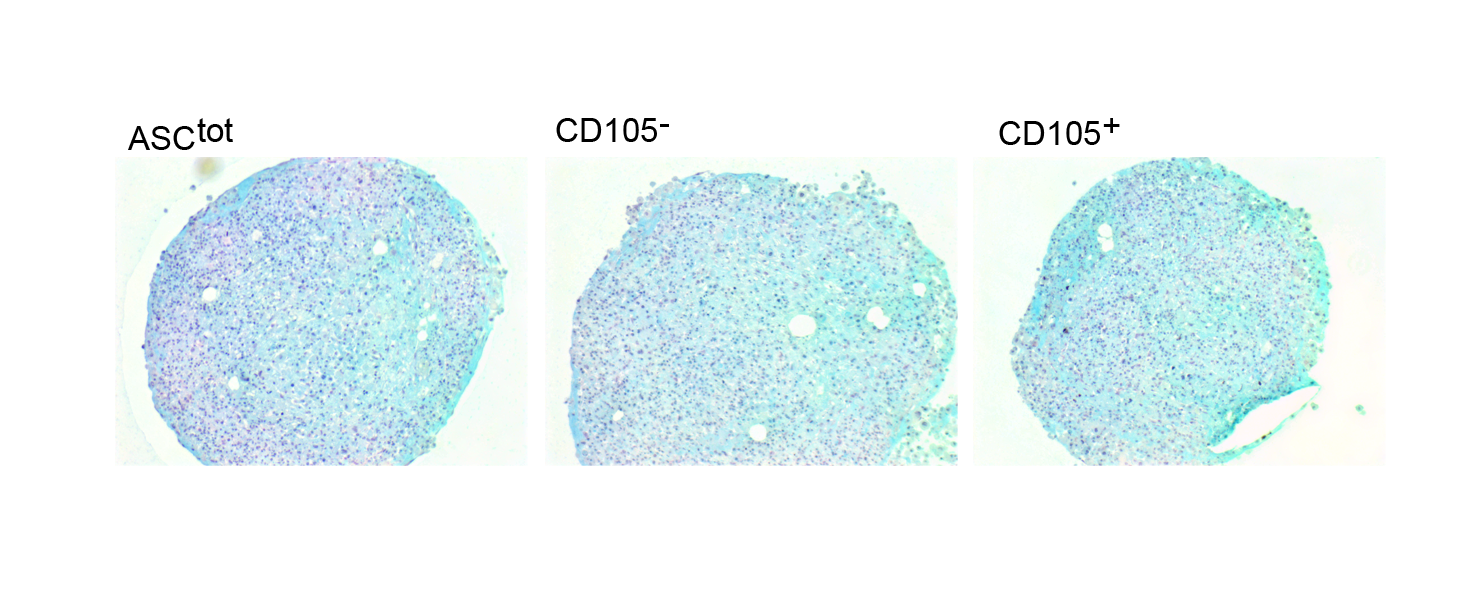

Supplement: Figure S4 — Chondrogenic potential of CD105- and CD105+ mASCs. Total ASCs or sorted CD105- and CD105+ mASCs (1 x 10^6^ cells/pellet) were resuspended in chondrogenic medium with TGF-β3 (20 ng/ml) and centrifuged to from a pellet. The medium was changed every 2-3 days. After 21 days, the pellets were fixed in 4% paraformaldehyde, embedded in paraffin, sectioned and stained for alcian blue. (TIF) [file pone.0076979.s004.tif]
